# Recent advances in pre-conditioned mesenchymal stem/stromal cell (MSCs) therapy in organ failure; a comprehensive review of preclinical studies

**DOI:** 10.1186/s13287-023-03374-9

**Published:** 2023-06-07

**Authors:** Mohammad Saeed Kahrizi, Elnaz Mousavi, Armin Khosravi, Sara Rahnama, Ali Salehi, Navid Nasrabadi, Farnoosh Ebrahimzadeh, Samira Jamali

**Affiliations:** 1 Department of Surgery, Alborz University of Medical Sciences, Karaj, Alborz Iran; 2 Department of Endodontics, School of Dentistry, Guilan University of Medical Sciences, Rasht, Iran; 3 Department of Periodontics, Dental School, Islamic Azad University, Isfahan (Khorasgan) Branch, Isfahan, Iran; 4 Department of Pediatric Dentistry, School of Dentistry, Semnan University of Medical Sciences, Semnan, Iran; 5 Department of Oral and Maxillofacial Radiology, School of Dentistry, Islamic Azad University, Isfahan (Khorasgan) Branch, Isfahan, Iran; 6 Department of Endodontics, School of Dentistry, Birjand University of Medical Sciences, Birjand, Iran; 7 Department of Internal Medicine, Faculty of Medicine, Mashhad University of Medical Sciences, Mashhad, Iran; 8 Department of Endodontics, Stomatological Hospital, College of Stomatology, Xi’an Jiaotong University, Shaanxi, People’s Republic of China

**Keywords:** Mesenchymal stem/stromal cells (MSCs), Pre-conditioning, Hypoxia, Organ failure, Transplantation

## Abstract

Mesenchymal stem/stromal cells (MSCs)‐based therapy brings the reassuring capability to regenerative medicine through their self‐renewal and multilineage potency. Also, they secret a diversity of mediators, which are complicated in moderation of deregulated immune responses, and yielding angiogenesis in vivo. Nonetheless, MSCs may lose biological performance after procurement and prolonged expansion in vitro. Also, following transplantation and migration to target tissue, they encounter a harsh milieu accompanied by death signals because of the lack of proper tensegrity structure between the cells and matrix. Accordingly, pre-conditioning of MSCs is strongly suggested to upgrade their performances in vivo, leading to more favored transplantation efficacy in regenerative medicine. Indeed, MSCs ex vivo pre-conditioning by hypoxia, inflammatory stimulus, or other factors/conditions may stimulate their survival, proliferation, migration, exosome secretion, and pro-angiogenic and anti-inflammatory characteristics in vivo. In this review, we deliver an overview of the pre-conditioning methods that are considered a strategy for improving the therapeutic efficacy of MSCs in organ failures, in particular, renal, heart, lung, and liver.

## Introduction

Researchers have focused on mesenchymal stem/stromal cells (MSCs) for the past 60 years because of their unique competencies, such as ease of isolation, lower immunogenicity, and immunoregulatory capacities [1]. These cells are highly amenable to cultivation in vitro; they can differentiate independently and secrete various growth factors and cytokines [2]. First, MSCs were procured from murine bone marrow (BM) by Friendenstein et al. and were called hematopoiesis-supporting cells in BM [3]. After that, Kaplan firstly proposes the term "mesenchymal stem cells," which are cells isolated from fully developed bone marrow (BM) that can usually differentiate into several types of mesenchymal origin cells [4]. Following the first successful human MSCs isolation from BM tissue [5], MSCs isolation from a diversity of adult tissues, such as the perivascular area, has been managed [6, 7]. Although there is no particular quantitative assay to provide MSCs identification in mixed cells population [8], the International Society for Cellular Therapy (ISCT) has provided minimum principles to determine MSCs. These criteria are the plastic adherence property, expressing CD73, D90, CD105 without CD14, CD34, CD45, and human leucocyte antigen-DR (HLA-DR) expression, and finally differentiation into adipocyte, chondrocyte, and osteoblast in vitro. The stromal vascular fraction of adipose tissue (AT) and BM are the two most common reservoirs of human MSCs [9]. However, the umbilical cord and the placenta, often discarded after delivery, are also excellent sources for human MSCs [10, 11]. Multiple types of cells, including adipose tissue, cartilage, bone, and even macrophages, have been shown to originate from MSCs [12, 13]. Importantly, MSCs have emerged as one of the most promising and vital potential sources for new clinical treatments for organ failure [14, 15].

Stem cell therapy has been the subject of many studies for its potential to cure many disorders. These include transplant infectious disease, progressive multiple sclerosis, diabetes, stroke, bronchopulmonary dysplasia, cardiomyopathy, and osteoarthritis [16]. Various in vivo reports indicated that MSCs could interfere with immune cells' infiltration, proliferation and activation post-transplantation [17, 18]. They also can inspire angiogenesis by direct differentiation, cell-to-cell interaction, or paracrine effects. Also, MSC-exosome contains cytokines, chemokines, microRNAs (miRNAs), growth factors, and proteins, making it an ideal therapeutic option [19]. According to these properties, they are an excellent candidate for treating organ failure, which is characterized by the inability of at least one of the body organs to conduct normal body functions [20]. However, natural MSCs in vivo survival and their biological effects on tissue recovery decrease with long-term cultivation called aging and also injected cells demonstrate poor targeted migration [21]. The harsh microenvironment with ischemia, inflammation, oxidative stress, and mechanical stress result in low survival rate of administrated cells [22]. Besides, MSC homing is inefficient, with only a small population of cells reaching the target tissue post systemic administration. These attritions signify a critical bottleneck in determining the full therapeutic competence of MSC-based therapies [23]. Thus, scientists have sought different modalities to bypass this drawback.

**Fig. 1:**
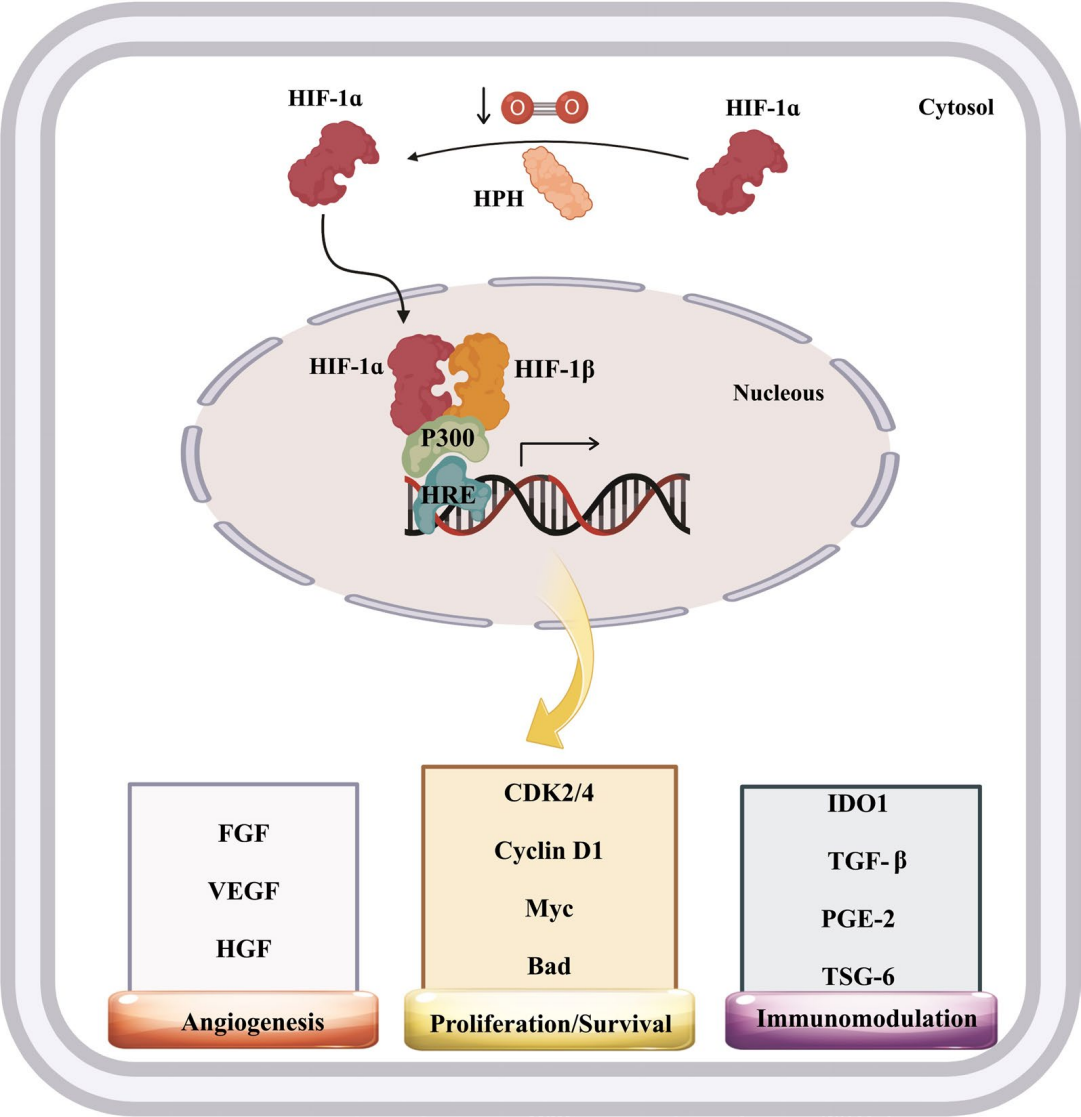
Hypoxia-inducible factor 1α (HIF-1α) signaling pathway. The figure depicts the action mechanism of HIF-1α in promoting the mesenchymal stem/stromal cells (MSCs)-mediated therapeutic influences. Hypoxia-response element (HRE), Hypoxia-inducible factor 1β (HIF-1β), CREB binding protein (CBP), HIF-1 prolyl hydroxylase (HPH), Indoleamine 2, 3-dioxygenase (IDO), Prostaglandin E2 (PGE2), Cyclin-dependent kinase (CDK), Vascular endothelial growth factor (VEGF), Hepatocyte growth factor (HGF), Fibroblast growth factor (FGF), Transforming growth factor beta (TGFβ), BCL-2 associated agonist of cell death (Bad), TNF-stimulated gene-6 (TSG-6)

In recent years, researchers have focused on designing or developing novel approaches to expand the therapeutic merits of MSCs [24]. In this light, pre-conditioning has engendered significant interest. Pre-conditioning is a method depending on a diversity of means to improve the potential of MSCs during ex vivo growth [25, 26]. Universally, pre-conditioning strategies comprise hypoxia, cell exposure with pharmacological/chemical agents or trophic factors/cytokines, pre-conditioning with physical factors, and finally, gene modification [27]. The pre-conditioning strategies, in turn, promote the various attributes of the, including their proliferative, secretory, migratory, pro-angiogenic, and anti-inflammatory aptitudes. These properties may bring about more preferred beneficial outcomes in vivo. For example, low O2 levels decrease the prolyl hydroxylation under hypoxic conditions, leading to hypoxia-inducible factor 1-alpha (HIF-1α) accumulation and nuclear translocation. In the nucleus, HIF-1α creates a heterodimer with HIF-1β and subsequently binds to the hypoxia-response element (HRE) in the target genes, allied with CBP/p300 (Fig. 1) [28, 29]. This assemblage adjusts the transcription of more than 70 genes, primarily complicated in angiogenesis, invasion/metastasis, survival, and proliferation (Table 1). Also, MSCs pre-treatment with carboxyl‐terminated hyperbranched polyester (CHBP) supports the mitochondria membrane potential (MMP) and mitochondrial membrane integrity in MSCs and also induces the Nrf2/Sirt3/FoxO3a pathway, thereby offering more resistance to oxidative stress [30].

**Table 1:** The effects of the hypoxia pre-conditioning on the gene expression profile of MSCs (preclinical studies)

Cell source	Origin	Target gene	Expression pattern	Results (ref)
BM	Human	Runx2 and TWIST	Down-regulation	Inhibited the osteogenic potential of the MSCs (in vitro) [174]
BM	Rat	VEGF and HGF	Up-regulation	Attenuated renal fibrosis (in vivo) [28]
BM	Rat	-	-	Improved renal function (in vivo) [131]
BM	Human	NLRP3 and caspase-1	Down-regulation	Reduced microglial pyroptosis following the intracerebral hemorrhage [175]
AT	Mouse	HLA-G, PGE2, and IDO	Up-regulation	Improved immunomodulation capacity [29]
BM				
WJ				
BM	Human	26S proteasome	Down-regulation	Increased immunogenicity [176]
AT	Human	p-Akt	Up-regulation	Improved angiogenic and anti-oxidative capacities (in vivo) [177]
BM	Porcine Human	VEGF	Up-regulation	More favored therapeutic characteristics [178]
		HMGB1, BCL-2 and caspase-3	Down-regulation	
BM	Human	PPARG	Down-regulation	Improved osteogenesis but inhibited adipogenesis of MSCs [179]
		HIF-1ɑ and RUNX2	Up-regulation	
Placental	Human	Cyclin A2/E1 and CDK2	Up-regulation	Improved proliferation [180]
		P21	Down-regulation	
BM	Mice	HIF-1α	Up-regulation	Induced MSC migration [181]
BM	Human	SDF-1 and VEGF	Up-regulation	Enhanced myogenesis under hypoxic conditions (in vitro) [182]
	Pig			
BM	Rat	COX-2	Down-regulation	Reduced immunoprivilege of allogeneic MSCs (in vitro and in vivo) [183]
Placental	Human	ERK, AKT, and CXCR4	Up-regulation	Improved migration and proliferation (in vitro) [184]
BM				
BM	Human	SUG1	Up-regulation	Improved the survival of transplanted cells (in vivo) [185]
	Rat			
BM	Rat	HIF-1α	Up-regulation	Enhanced viability and reduced apoptosis [186]
BM	Mouse	ALP, RUNX2, COL1A, and OCN	Up-regulation	Improved proliferation and osteogenic differentiation (in vitro) [187]
BM	Mouse	HIF-1α and Akt	Up-regulation	Improved proliferation and antioxidant activity [188]
BM	Human	HLA-DRα	Up-regulation	Reduced immunoprivilege of MSCs (in vivo) [189]
BM	Human	Akt	Up-regulation	Improved chondrogenesis and inhibited terminal differentiation by inducing the PI3K/Akt/FoxO pathway [190]
ESCs	Human	PDGF-BB, IGFBP-6, VEGF-A, and angiogenin	Up-regulation	Improved viability (in vivo) [191]
AT	Human	VEGF	Up-regulation	Enhance angiogenesis by PKA signaling pathway (in vitro) [192]
UC	Equine	SOX2, OCT4, and Nanog	Up-regulation	Enhanced stemness of MSCs [193]
BM	Mouse	HIF-1α	Up-regulation	Increase MSC proliferation and long-term survival post-irradiation [194]
UCB	Human	HIF-1α	Up-regulation	Improved expansion [195]
Olfactory mucosa	Human	p16INK4A, p21, and p53	Down-regulation	Reduced senescence [196]

Mesenchymal stem/stromal cells (MSCs), Adipose tissue (AT), Bone marrow (BM), Umbilical cord (UC), Umbilical cord blood (UCB), Wharton's jelly (WJ), Embryonic stem cells (ESCs), Runt-related transcription factor 2 (Runx2), Twist-related protein-1 (TWIST), Vascular endothelial growth factor (VEGF), Hepatocyte growth factor (HGF), NLR family pyrin domain containing 3 (NLRP3), Indoleamine 2, 3-dioxygenase (IDO), Prostaglandin E2 (PGE2), Human leukocyte antigen G (HLA-G), High mobility group box 1 (HMGB1), B cell lymphoma 2 (BCL-2), Hypoxia-inducible factor (HIF), Peroxisome proliferator-activated receptor gamma (PPARG), Cyclin-dependent kinase 2 (CDK2), Stromal cell-derived factor 1 (SDF-1), Cyclooxygenase 2 (COX-2), Extracellular signal-regulated kinases (ERK), C-X-C chemokine receptor type 4 (CXCR4), Osteocalcin (OCN), Protease regulatory subunit 8 homolog (SUG1), Alkaline phosphatase (ALP), Collagen type IA (COL1A), Platelet-derived growth factors (PDGF), Insulin-like growth factor-binding protein 6 (IGFBP-6), SRY-box 2 (SOX2), Pour octamer-binding transcription factor 4 (OCT4), Protein kinase A (PKA), Phosphoinositide 3-kinases (PI3Ks), Forkhead box O (FOXO)

Herein, we will look into the use of pre-conditioned MSCs in organ failure to deliver a unified and comprehensive view of the best approach to augment the therapeutic influences of MSCs in these conditions, with a particular focus on the recent preclinical reports.

## The MSCs sources and their differences

Stem/progenitor cells with MSC-like biological features have been detected in different mature tissues in the past decade, including the BM, skin, placenta, umbilical cord blood (UCB), umbilical cord tissue, adipose tissue (AT), dental pulp, infant teeth, testicles, brain, etc. With mounting evidence that MSCs isolated from different sources form a diverse cell population, the development of uniform criteria for identifying MSCs became an urgent necessity [31].

Expression of CD73, CD90, and CD105 constitute the minimum criteria for identifying tissue-isolated MSCs [32]. The hBM-MSCs, hAT-MSCs, human adipose-derived stromal cells (hADSSCs), and human muscle-derived progenitor cells (hMDPCs) all express these markers at high levels [17, 33–35]. These cells also lack the hematopoietic markers CD34 and CD45. In this light, CD146 is a second MSC marker expressed by MSCs from different sources [17, 31]. Also, MSCs from various sources have varying degrees of paracrine potential, significantly impacting their aptitude to influence target cells and either dampen or amplify the immune response. MSCs derived from BM and secrete cytokines and growth factors such as interleukin (IL)-6, IL-8, monocyte chemoattractant protein-1 (MCP-1), vascular endothelial growth factor (VEGF), osteoprotegerin, and tissue inhibitor of metalloproteinases 2 (TIMP2) [36]. Also, increased levels of the interferon-gamma (INF-γ), platelet-derived growth factor A (PDGFA), VEGF, IL-10, and stromal-derived factor (SDF) were found in human exfoliated deciduous teeth (SHED) in comparison to Wharton's jelly (WJ)- and BM-MSC [37]. Significantly, the microenvironment of MSCs affects the paracrine abilities of stem/progenitor cells. Interestingly, compared to other sources, skin-derived MSCs can secrete higher levels of trophic substances such as VEGF, granulocyte colony-stimulating factor (G-CSF), hepatocyte growth factor (HGF-1), and basic fibroblast growth factor (bFGF) [38]. Ribeiron et al. also found that AT-MSCs inhibited NK and B cells more effectively than BM- and UCB-MSCs [39]. Furthermore, compared to UC-MSCs, AT-MSCs showed more significant inhibitory effects on serum IL-1, IL-6, and IL-8 levels in lipopolysaccharide (LPS)-treated mice [40]. UC-MSCs also have demonstrated more evident proliferation and clonality due to the reduced expression of p53, p21, and p16 compared to cells derived from BM and AT [40]. In another study, BM-MSCs and WJ-MSC showed superiority over AT-MSCs in terms of proliferation and clonality potential [41]. In addition, AT-MSCs and UC-MSCs can demonstrate more prominent osteogenic potential compared to chorionic membrane (CM)- and decidua (DC)-MSCs [42].

Taken together, while MSCs from various tissues share many traits, their biological activity and some markers vary depending on the tissue from which they were derived. For researchers interested in the use of MSCs in clinical settings, understanding the biological principles underlying MSCs should be a key factor. For instance, higher CD146 expression promotes the cells migration capability in vitro and in vivo, and its down-regulation has correlation with higher osteogenic capacity [43]. These proofs verify differences between the MSCs from various sources, highlighting the importance of determining better sources respecting the conditions.

## MSCs' rationality for treatment of organ failure

In vitro, MSCs can differentiate into numerous mesoderm lineages and differentiated cells, such as osteoblast, fats, skeletal muscle myocytes/myotubes, pancreatic islet cells, and cardiomyocytes, when grown in a growth factor-rich culture environment [31]. However, small populations of MSCs differentiate into functional cells in vivo [44, 45]. To influence other cells, MSCs produce exosomes and micro-vesicles that carry potent angiogenic mediators, cytokines, or mRNA molecules [46]. The process by which MSCs are released from BM is critically vital to their regenerative function. These cells reside primarily in BM but can be found in other organs and tissues because of their mobile nature. Elm et al. detected the presence of MSCs in the PB of people who had suffered hip fractures [47]. Based on their observations, MSCs were found in peripheral blood (PB) from 22% of hip fracture patients, 46% of younger fracture patients, and in none of 63 pre- and postmenopausal women with hip OA [47]. Meanwhile, several lines of evidence suggest that MSCs are secreted from the BM in response to systemic cues; hypoxia recruits MSCs to the PB, triggering liver injury. Also, MSCs could be released from adipose tissue in response to inflammation and then collected in lymph nodes and blood arteries [48]. Recent research has demonstrated the importance of CCR9, CXCR4, and c-MET in guiding endogenous MSC migration to the damaged liver [49]. Further, MSCs have garnered interest as they could promote tissue regeneration and homeostasis in inflammatory conditions such as graft-versus-host disease (GVHD), multiple sclerosis (MS), lung inflammation, arthritis, and Crohn's disease (CD) [50, 51]. Exogenous MSCs are frequently applied to bring about tissue recovery in vivo due to their anti-inflammatory properties and their capacity to provoke angiogenesis and boost the proliferation of damaged cells [52–55].

### Inhibition of inflammation

Recent years have seen remarkable progress in our knowledge of how MSCs modulate the immune system and reduce inflammation. MSC responses may vary with the intensity of environmental cues. In the earliest stages of inflammation, MSCs amplify the inflammatory response by sensing pro-inflammatory signals through IL-1-receptors (IL-1Rs), IFN-receptors (IFNRs), toll-like receptors (TLRs), and TNF- receptors (TNFRs) [56]. They increase T cell activation by secreting chemokines like C-X-C motif ligand (CXCL)-9, macrophage inflammatory protein-1 (MIP-1), CCL5, and CCL10. Low levels of inflammatory signals like TNF-ɑ and IFN-γ enhance the rise in chemokine secretion at this time [57, 58]. In later phases, when pro-inflammatory molecules like IL-1, IFN-γ, and TNF-α are present in more significant concentrations, MSCs are activated, and then secrete TGF-β and IL-10 to bypass inflammation and halt autoimmune responses [59]. Indoleamine 2,3-dioxygenase (IDO) and inducible nitric oxide synthase (iNOS) decrease the proliferation, migration, and maturation of dendritic cells (DC) and T cells and thus bargain their ability to deliver antigens. Therefore, IDO or iNOS levels may determine the pro-inflammatory or anti-inflammatory effects of MSCs [60]. Additional studies show that CD5 + regulatory B cells protect against colitis when treated with human MSCs, CD23 + CD43 + B cells and MSCs generated from human umbilical cords [61]. Each of these cells play a role in reducing intestinal inflammation [61]. Therefore, MSCs may suppress inflammation by enhancing anti-inflammatory factors and decreasing pro-inflammatory mediators [62, 63]. When MSCs come into direct contact with cells, they can dampen immune responses [61]. For instance, studies in rodents with GVHD exhibited that systemic injection of human MSC-exosome improved animals' survival by inhibiting CD4^+^ and CD8^+^ T cell performance and infiltration and increasing Treg cell activity [64]. In addition, TNF-α, NF-κB, IL-6, and IL-8 levels were reduced in the lung tissue of animals with acute lung injury upon MSCs systemic administration [65].

### Induction of angiogenesis

Impaired angiogenesis and endothelial dysfunction are probably involved in the augmented prevalence of organ dysfunction. Angiogenesis is required for tissue repair, and a sufficient vascular network is paramount to supply blood and growth factors to damaged tissues [66]. Because of the marked positive effect on angiogenesis, MSCs have significant therapeutic power for treating organ failure such as heart failure (HF). Of course, the application of MSC-based therapies is confined by their low persistence level in targeted tissues and the low capabilities of transdifferentiation in vivo [67]. The most crucial property of MSCs for treating ischemic diseases is the secretion of pro-angiogenic mediators like VEGF, HGF, and FGF and their differentiation potential into vascular phenotypes in vitro [68]. They can promote endogenous angiogenesis via microenvironmental modulation and differentiating into various types of vascular cells [69]. Some proofs demonstrated that MSCs could be injected into injured areas and develop into the heart and endothelial cells [70]. Further, some clinical investigations indicate that MSCs can ameliorate key clinical parameters in patients suffering from organ failure [71]. MSCs can stimulate organ normal function by inducing angiogenesis through the secretion of VEGF, macrophage colony-stimulating factor (MCF), and IL-6. Meanwhile, VEGF serves essential roles in angiogenesis and microvascular permeability. Interaction between VEGF/VEGFR signaling in endothelial cells (EC) facilitates the production of cytokines and chemokines and up-regulates the cell adhesion molecules expression [72]. To promote local angiogenesis, MSCs can secrete both hepatocyte growth factor (HGF) and stromal cell-derived factor 1 (SDF-1) [73, 74]. SDF-1 is a critical chemokine that can regulate various physiological processes, including stimulating the proliferation of ECs and generating capillary tubes [75]. The ECs express the receptor c-Met, by which HGF exerts its angiogenic effect by tyrosine phosphorylation. The therapeutic efficacy of HGF was studied in a clinical experiment, offering some benefits in organ ischemia [76, 77].

### Enhancing target cell proliferation and differentiation

Replacing damaged cells is needed for ameliorating organ dysfunction. Human MSCs are a great source of cells for cell transplantation and tissue engineering because of their capacity to stimulate target cell proliferation. For instance, MSCs secreted IGF-1 can promote primary hepatocyte proliferation [78]. In co-culture conditions, MSCs enhanced the numbers of the proliferating cell nuclear antigen (PCNA) expressing hepatocyte in vitro [79]. Likewise, MSCs-derived exosomes intensified cardiomyocyte proliferation by miR-210 delivery [80]. The miR-210 overexpressing MSC-exosomes also could improve myocyte protection in response to both in vitro and in vivo stress [80]. Exosomal miR-25-3p from MSCs was capable of decreasing cardiomyocytes apoptosis and sustaining their expansion by negative regulation of enhancer of zeste homolog 2 (EZH2) [81]. In addition, Yi et al. found that miR-30b-3p-overexpressing MSCs increased type II alveolar epithelial cells (AECs) growth and protected versus lipopolysaccharide-induced lung damages by inhibiting serum amyloid A 3 (Saa3) [82].

## The rationality of MSCs pre-conditioning

**Fig. 2:**
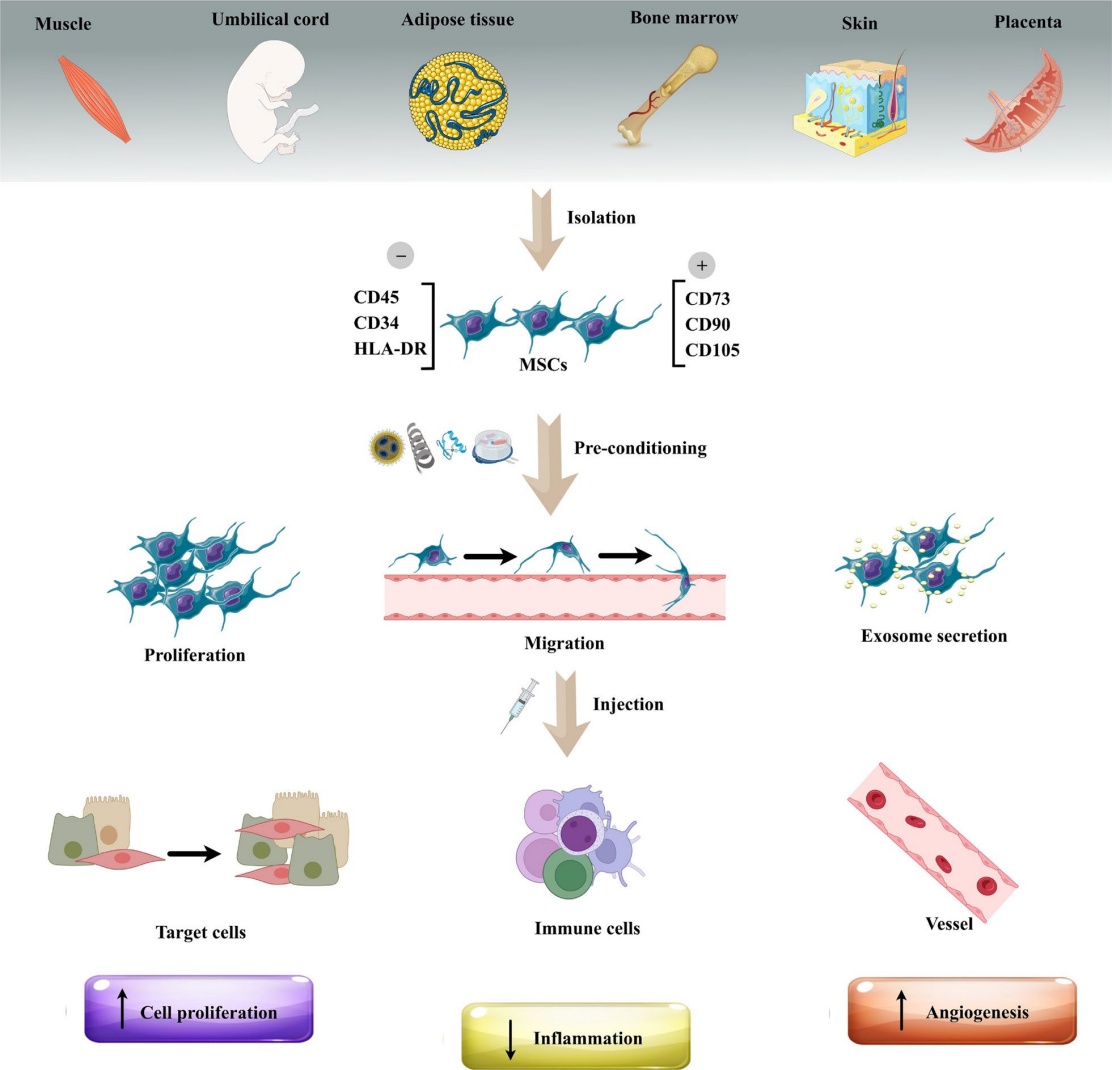
The rationality of the pre-conditioning of mesenchymal stem/stromal cells (MSCs). Pre-conditioning, such as exposure to specific ingredients or biomolecules and genetic modification of MSC, improves MSC function in vitro and in vivo

Cultural conditions are the most imperative factors influencing the functional potential of MSCs. Regeneration functions of MSCs and their clinical implementation for repairing and regenerating damaged and destroyed tissues are also hindered by "disease conditions" and the "age" of the donor. Accordingly, stem cells are suggested to be manipulated before their use in clinical settings to potentiate their survival, migration, and therapeutic competencies in vivo [83]. Pre-conditioning cells in a particular design/engineering with varied physical or chemical characteristics and variables under ex vivo settings has increased MSCs' capability to survive in hostile microenvironments and boost their immune responses [84]. Several methods, such as low-heat shock, glucose depletion, and pre-conditioning with growth factors, have been employed to accomplish this. Since oxygen levels are already low in stem cell niches compared to typical situations, hypoxic pre-conditioning may improve their natural capabilities [85]. Adapting cells to their external environment, reducing oxidative stress, switching metabolism to glycolysis, increasing cell proliferation, differentiation, and stemness maintenance, and increasing their movement to sustain hypoxic conditions after transplantation all suggest that hypoxia may be a valuable strategy for improving cell functions [83]. Hypoxic pre-condition up-regulates anti-apoptotic proteins expression in MSCs and thus promotes their survival in the hostile environment. Hypoxic pre-conditioning also reduces MSCs' glucose consumption, lactate release, and cytochrome c and heme oxygenase-1 (HO-1) levels [86]. Further, the human MSCs' exposure to IFN-γ could ease the inhibition of NK activation and improve the protection of MSCs from NK-induced cytotoxicity [87]. Besides, 3-dimensional cell culture could intensify the immunomodulatory aptitudes of human MSCs, as shown by reduced TNF-α, IL-6, IL-12p40, IL-23, and CXCL2 and improved IL-10 levels in conditioned media [88]. Recent reports also indicated that MSCs pre-treatment with angiotensin II enhances the outcome of MSC-based therapy for myocardial infarction (MI) in part via increasing the paracrine production of VEGF, and supporting gap junctions (GJs) [89]. The positive effects of the MSCs on angiogenesis could also be further heightened by hypoxia pre-treatment as a result of the increased secretion of VEGF [90]. Figure 2 depicts the effects of the pre-conditioning on MSCs' therapeutic benefits in vivo. As described, pre-conditioned MSCs show better therapeutic efficacy over naïve MSCs concerning the. They raise target cell growth, persuade angiogenesis and modify immune responses.

## Pre-conditioned MSCs in lung failure

Lung failure is the most shared organ failure seen in the intensive care unit. The pathogenesis of acute respiratory failure (ARF) can be categorized as (1) neuromuscular in origin, (2) secondary to acute and chronic obstructive airway disorders, (3) alveolar procedures like cardiogenic and noncardiogenic pulmonary edema and pneumonia, and (4) finally vascular disorders such as acute or chronic pulmonary embolism [91, 92]. Based on the literature, MSCs and their secreted products can attenuate lung inflammation and support its structure and performance [93, 94]. The safety, feasibility and efficacy of MSCs administration is under-investigation in phase 1 and phases 2 trials in patients with lung failure and related conditions (NCT02112500, NCT04392778 and NCT04537351).

As previously described, a growing body of reports has signified that hypoxia, thermal shock, small-molecule medicines, cytokines and growth factors could increase the therapeutic merits of MSCs transplantation [96–98]. Genetic modification and overexpression of pro-survival genes, chemokine receptors, or anti-apoptotic proteins can also be used to perform cellular pre-conditioning before transplantation [99–102].

In 2019, Chen et al. found that BM-MSCs overexpressing heme oxygenase-1 (HO-1) could alleviate lipopolysaccharide (LPS)-induced acute lung injury (ALI) and resultant lung failure in rats [103]. The HO-1 has antioxidant, anti-inflammatory, and anti-apoptotic properties [103]. The release of HO-1 by MSCs post-transplantation has been shown to elicit protective effects against ALI. Compared to parental MSCs, MSCs-HO-1 transplantation showed significant improvements in cell survival, apoptosis, and paracrine activity in vivo [103]. Further, MSCs-HO-1 exhibited more evident pro-survival and anti-apoptotic impacts and paracrine activity in vitro. These findings shed light on the potential of genetic engineering of MSC for managing ALI [103]. Likewise, systemic injection of manganese superoxide dismutase (MnSOD)-overexpressing MSCs led to reduced lung inflammation, as shown by decreased IL-1, IL-6, and TNF-α levels [104]. Importantly, MnSOD-MSCs differentiated into epithelial-like cells in vivo [104], indicating the excellent capability of MnSOD-MSCs. Also, Liao et al. (2023) have found that administration of IL18-hUC-MSCs could drastically decrease viral load, fibrosis, and cell apoptosis in acute lung injuries [105]. Notably, T cell exudation and pro-inflammatory cytokine release in bronchoalveolar lavage fluid (BALF) were significantly inhibited by IL18-hUCMSC therapy [105].

## Pre-conditioned MSCs in heart failure

Heart failure (HF) is a clinical syndrome characterized by structural and functional failings in the myocardium, eventually weakening ventricular filling or the ejection of blood. The HF often results from poor left ventricular function [106]. Decreased diastolic filling and ejection fraction can both result in less blood leaving the heart into systemic circulation [107]. Of course, deficits in the pericardium, myocardium, endocardium, heart valves, or great vessels alone or in combination are also allied with HF. Over the past two decades, numerous investigations have been carried out on the potential of MSCs for cardiac cell regeneration [108, 109]. Several MSCs-based strategies have been studied by employing the three ways of direct differentiation to heart cells, differentiation to vascular cells, and paracrine signaling [110]. The safety and modest efficacy of UCB-MSCs systemic administration has been verified in patients with HF [111]. Improvements in left ventricular function, functional status, and quality of life were detected in treated patients [111]. BM-MSCs transplantation by intra-myocardial [112] and intra-coronary route [113] also were safe and led to increased myocardial function in patients with HF.

In vitro, hypoxia pre-conditioning boosts hUC-MSCs proliferation and enhances their differentiation into cardiomyocyte-like cells (CLCs) [114]. Besides, it has previously been found that the growth arrest of particular gene 6 (Gas6) influences cell growth, adhesion, chemotaxis, mitogenesis, and cell survival because of the presence of gamma-linolenic acid-carboxyglutamic acid (Gla) [115]. Functional studies suggest that Gas6 overexpression could significantly reduce MSC apoptosis and increase MSC survival in vitro and in HF animal models compared to naïve MSCs. Also, Gas6 could enhance VEGF, bFGF, SDF, and IGF-1 secretion from MSCs [116]. Likewise, HIF-1-overexpressing MSCs were found to increase cardiac output and decrease the size of myocardial scars in HF in vivo models [117]. Further, HIF-1 overexpression significantly augmented the secretion of angiogenesis proteins like activin A, angiopoietin, artemin, endothelin-1, MCP-1, and remodeling factors ADAMTS1, FGFs, TGF-β, MMPs, and serpins in MSCs in vitro [117]. Genetically modified MSCs to overexpress VEGF in hypoxic conditions also increased myocardial neovascularization in ischemic heart disease [118]. These engineered MSCs also decreased the apoptotic cell numbers in the infarcted area and caused the reduction of left ventricular remodeling in vivo [118]. Besides, another study on a mouse model of heart failure demonstrated that overexpression of anti-fibrotic substances, adrenomedullin (ADM), dramatically improved heart function, decreased fibrotic area, and decreased MMP-2 expression [119–122]. The ADM-MSC-treated group also shows markedly higher MSCs survival after transplantation. These findings indicate that MSCs overexpressing ADM can potentially increase anti-fibrotic actions, improving heart function in animals with heart failure [119]. Finally, pre-conditioning MSCs with caspase inhibition and hyperoxia could boost their capacity to diminish left ventricular remodeling and sustain left ventricular activity [123]. Additionally, gene and protein expression of caspases 1, 3, 6, 7, and 9 were decreased drastically in MSCs pre-conditioned with hyperoxia, caspase inhibition, or both, while up-regulating Akt1, NF-κB, and Bcl-2 expression in pre-conditioned MSCs. These alterations ultimately led to a substantial increase in MSC proliferation in hypoxic environment in vivo [123].

## Pre-conditioned MSCs in renal failure

The term renal failure means incapability of the kidneys to accomplish the excretory activity, driving retention of nitrogenous waste yields from the blood. Once a patient necessities renal replacement therapy, the ailment is named end-stage renal disease (ESRD) [124]. Although kidney transplantation is now the gold standard for treating ESRD, significant difficulties exist in this field, particularly in preventing transplant rejection and ensuring long-term organ acceptance. In recent years, the probability of acute rejection (AR) has been mitigated by using triple immunosuppressive medication [125]. Given their involvement in regulating the immune system, MSCs have emerged as a promising candidate in this context [85]. Recently, Shao et al. (2021) exhibited that intravenous administration of autologous BM-MSCs led to improvement in renal and systemic functional parameters from baseline in Chinese renal failure patients [126].

One of the most prevalent injuries sustained with a kidney transplant is ischemia/reperfusion (I/R) damage. As a result of their ability to heal cellular damage, reduce tissue rejection, and attain organ tolerance, MSCs are a promising cell therapy candidate for use in kidney transplantation [127]. The MSC infusion in kidney transplant recipients is feasible, permits enlargement of Treg in the peripheral blood, and regulates memory CD8 + T cell function [128]. The pre-conditioning of MSCs also is believed to potentiate parental MSCs capability to support successful kidney transplantation by increasing the survival of MSCs and potentiating their migration and protecting them from natural killer (NK)-mediated cytotoxicity [129]. In this light, MSCs treatment with melatonin prior administration was shown to boost the survival of MSCs, enhance cell proliferation and angiogenesis, and enable quicker recovery of the renal function [130].

A recent study in an animal model of gentamicin-induced acute renal failure (ARF) showed that MSCs pre-conditioned with hypoxia could induce a more suitable therapeutic effect than naïve MSCs [131]. Hypoxia-induced MSCs administration diminished blood urea nitrogen (BUN) and creatinine level, thus supporting renal function [131]. The histological analysis of renal tissue isolated from hypoxia-induced MSCs treated animals also verified these findings [131]. Additionally, miR-19a-3p and miR-20a-5p co-expressing human iPS-MSCs protected kidney function in rat models of chronic kidney disease following acute ischemia [132]. Further, genetically modified iPS-MSCs were capable of decreasing oxidative stress, inflammatory downstream signaling, and renal cell death in vitro [132]. Likewise, Cao et al. (2021) showed that miRNA-133b-overexpressing MSCs could attenuate renal fibrosis in an animal model of renal failure in part by inhibition of connective tissue growth factor (CTGF) expression in renal tissue [133]. Negative regulation of CTGF leads to the suppression of the TGF-β1-induced EMT of HK2 cells, a proximal tubular cell (PTC) line derived from normal kidney, in vitro [133]. Nonetheless, genetic modification of MSCs to overexpress CXCR4 and CXCR7 did not increase their homing therapeutic capacities in acute kidney injury in vivo models [134]. Also, scientists found that administration of neither native nor engineered MSCs amended renal failure in vivo [134]. In contrast, Liu et al. (2013) demonstrated that CXCR4 overexpression increased BM-MSCs migration to the kidney tissue in acute kidney injury [135]. The SDF-1/CXCR4 signaling plays a central role in this event by transducing the PI3K/AKT and MAPK in BM-MSCs [135]. Besides, it has been suggested that expanding MSCs in hollow fiber bioreactor-based 3D) culture systems could potentiate their ability to ameliorate renal function in vivo mainly by enhancing exosome secretion [136].

## Pre-conditioned MSCs in liver failure

The liver is a crucial organ that aids in digestion, elimination of toxins, and immune system function. The liver can renew itself because it contains particular cells, including mature liver cells, intrahepatic stem cells, and extra stem cells [137]. Although endogenous regeneration is possible, it is not effective after severe damage. Infections, medicines, toxins, chemicals, autoimmune disease and metabolic diseases are the leading causes of acute liver failure (ALF), in which liver dysfunction produces severe damage and necrosis. Acute liver failure has a high fatality rate despite aggressive treatment [138]. Liver transplantation has become less effective as the primary treatment for liver illnesses due to a lack of organ donors, unfavorable effects of immunosuppressive medicines on recipients, and procedural issues [139]. A shortage of oxygen and the presence of radical oxygen species (ROS) cause the vast majority of transplanted stem cells to die just a few days after administration. Investigations suggest that MSCs have a higher capacity to restore damaged liver tissue due to their ability to develop into specialized cells when incubated with damaged liver cells. Recovery of liver enzymes and histological improvement due to central necrosis repair has been documented [140, 141].

Various clinical trials have evidenced the safety and feasibility of MSCs along with enhanced serum albumin, cholinesterase, and prothrombin activity in patients with liver failure [142–145].

Notwithstanding, because of the limited success of MSCs in liver diseases therapy, numerous studies have been done to address this issue [146–149].

IL-1 is a therapeutic option for sustaining MSCs to treat ALF by promoting the MSCs' capacity to regenerate damaged liver [150]. Through the increasing CXCR4 expression and ensuing enhancement in MSCs homing capacity, IL-1 pre-treatment can improve MSCs-mediated impacts on ALF [151]. Also, direct modification of MSCs to overexpress CXCR4 potentiates their potential to increase liver regeneration [152]. Also, sodium butyrate (NaB) treatment was supposed to improve the hepatic differentiation of BM-MSCs post-transplantation in vivo [153]. The NaB-MSCs transplantation also enhanced albumin (ALB), alpha 1-antitrypsin (AAT), and the serum total protein (TP), while reducing serum alanine transaminase (ALT) levels in vivo [153]. Further, umbilical cord blood (UCB)-MSCs engineered to overexpress the VEGF _165_ gene could facilitate ALF treatment. VEGF_165_ overexpression promoted the multipotency of UCB-MSCs and increased their homing and colonization in the liver tissues of ALF rat [154]. VEGF_165_ –MSCs transplantation ameliorated liver damage and improved liver regeneration more evidently than native UCB-MSCs [154].

Interleukin-35 (IL-35) is an emerging cytokine critical for preventing autoimmune illnesses and responsible for the Treg's ability to moderate and decrease immunological responses [155]. The IL-35 gene-modified MSCs could migrate to the damaged liver tissues, reduce hepatocyte apoptosis, and down-regulate IFN-γ secretion by liver mononuclear cells mainly by negative regulation of JAK1-STAT1/STAT4 axis by IL-35 [156].

**Fig. 3:**
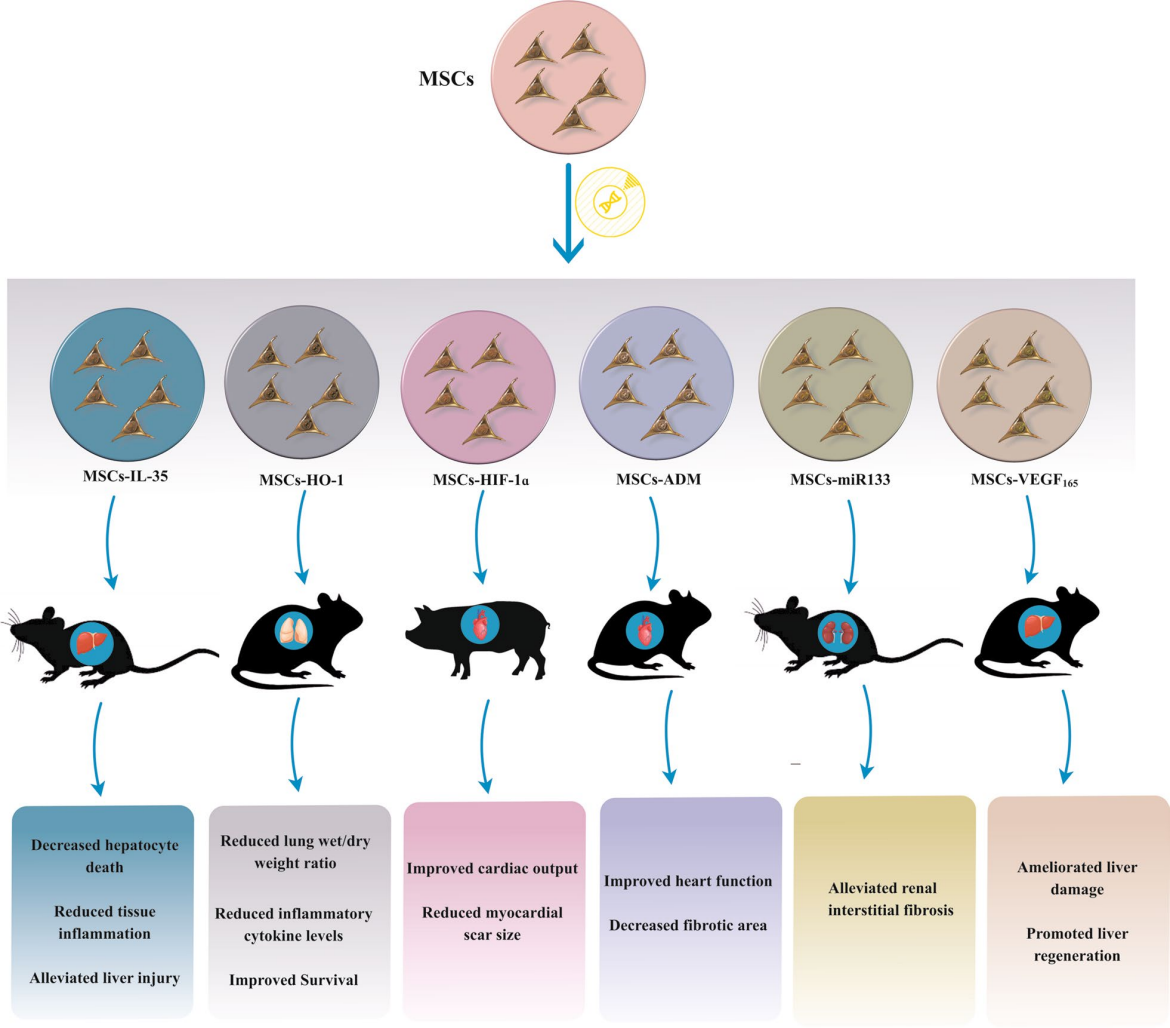
The application of genetically modified mesenchymal stem/stromal cells (MSCs) in organ failure. Adrenomedullin (ADM), Heme oxygenase-1 (HO-1), Vascular endothelial growth factor 165 (VEGF_165_), Hypoxia-inducible factor 1α (HIF-1α), MicroRNA133 (miR-133), Interleukin-35 (IL-35)

A summary of the studies investigating the therapeutic effects of genetically modified MSCs in organ failure disease is provided in Table 2. Figure 3 also depicts the impact of the HIF-1ɑ-, ADM-, miR-133-, IL-35-, VEGF_165_-, and HO-1-overexpressing MSCs in vivo.

**Table 2:** Genetically modified MSCs in organ failure and related conditions (preclinical studies)

Condition	Cell Source	Gene	Study type	Results (ref)
Liver failure	UCB	VEGF_65_	In vivo (rat)	Stimulation of substantial therapeutic influences on ALF [154]
Heart failure	BM	ADM	In vivo (rat)	Enhanced heart function and decreased fibrotic area volume and MMPs levels in heart tissue [119]
Heart failure	BM	HGF	In vivo (rat)	Improved LV systolic and diastolic function [197]
Heart failure	BM	VEGF	In vivo (swine)	Enhanced neovascularization, reduced hypertrophy, potentiated myocardial bioenergetic characteristics, and contractile function [198]
Renal failure	BM	IDO	In vivo (mice)	Regeneration of the renal tissue by adjusting the polarization of the macrophage [199]
Lung failure	BM	HO-1	In vitro	Improved pro-survival, anti-apoptotic, and paracrine functions of MSCs-HO-1 [103]
			In vivo (rat)	
Liver failure	UC	HNF4α	In vivo (mice)	Eliciting the marked therapeutic influences on ALF [200]
Heart failure	BM	ILK	In vivo (swine)	Ameliorating the ventricular remodeling and cardiac activity [201]
Heart failure	BM	Gas6	In vivo (rat)	Eliciting functional recovery [116]
Liver failure	BM	CXCR4	In vivo (mice)	Enhanced migration and ameliorated tissue damage by stimulating hepatoprotective influences [152]
Heart failure	AT	Myocardin	In vitro	Enhanced myogenic marker expression, blood flow as well as arteriogenesis [202]
	BM		In vivo (mice)	
Heart failure	BM	VEGF	In vivo (rat)	Reduced cardiomyocyte cell apoptosis in vitro and marked reduction of LV remodeling [118]
Renal failure	iPS	miR-19a miR-20a	In vitro	Improved renal function [132]
			In vivo (rat)	
Renal failure	UC	IGF-1	In vivo (rat)	Ameliorated biochemical variables in serum or urine related to renal function [203]
Renal failure	BM	TGF-β1	In vivo (rat)	Improved renal ischemic reperfusion injury (IRI) by targeting the CXCR4 expression on cell membranes [204]
Liver failure	AF	IL-1R antagonist	In vivo (rat)	Enhanced liver function and prolonged survival [205]
Ovarian failure	BM	miR-21	In vivo (rat)	Restoring ovarian function by decreasing granulosa cell apoptosis [165]

Mesenchymal stem/stromal cells (MSCs), Adipose tissue (AT), Bone marrow (BM), Umbilical cord (UC), Umbilical cord blood (UCB), induced pluripotent stem cells (iPSCs), Amniotic fluid (AF), Embryonic stem cells (ESCs), Vascular endothelial growth factor (VEGF), Hepatocyte growth factor (HGF), Indoleamine 2, 3-dioxygenase (IDO), Adrenomedullin (ADM), Heme oxygenase-1 (HO-1), Hepatocyte nuclear factor 4 alpha (HNF4A), Integrin-linked kinase (ILK), Growth arrest-specific gene 6 (Gas6), C-X-C chemokine receptor type 4 (CXCR4), Insulin-like growth factor (IGF)-1, Transforming growth factor beta 1 (TGF-β1), Interleukin-1 receptor (IL-1R), Acute liver failure (ALF), Matrix metalloproteinases (MMPs), Left ventricular (LV)

## Pre-conditioned MSCs in ovarian failure

One of the common disorders affecting women that contributes to 1% of female infertility is premature ovarian failure (POF) [157]. Hypoestrogenism, or a lack of estrogen, an elevated gonadotropin level, and, most significantly, amenorrhea are all clinical signs of POF. As the most popular hormone replacement therapy cannot successfully restore ovarian function [158], there is now a greater need for effective and novel POF therapeutics. Meanwhile, human MSCs therapy offers new opportunities for POF as regenerative medicine advances [159, 160]. In mice receiving chemotherapy, the MSCs therapy was discovered to diminish granulosa cell (GC) apoptosis and DNA damage [161] and can also promote the growth of primordial follicles and raise FSH levels to levels that are close to normal [162]. MSCs also promote reactivate folliculogenesis [163] and increase insulin-like growth factor-1 (IGF-1) in ovaries [164].

Recent studies demonstrated that overexpressing miR-21 in BM-MSCs could restore ovarian function in rats with chemotherapy-induced POF. This was associated with the inhibition of granulosa cell apoptosis by targeting recombinant human programmed cell death 4 (PDCD4) and phosphatase and tensin homolog deleted on chromosome 10 (PTEN) [165]. Numerous reports also have shown that heat shock (HS) pre-treatment can cause the production of heat shock transcription factor (HSF1), which activates particular signaling pathways (e. g., HSF1/miR-34a/HSP70) to create a number of HSPs [166]. HSPs play a role in the obstruction of various apoptotic pathways. Apoptosome formation and the mitochondrial apoptotic pathway, for instance, are blocked when HSP27 and HSP90 bind to Apaf-1 [167]. In order to prevent the caspase-mediated apoptotic pathway from being activated, HSP70 interacts with apoptosis inducing factor (AIF) [168]. By inhibiting granulosa cell apoptosis more effectively than with naive MSC therapy in the rat model of chemotherapy-induced POF, the HS pre-treatment of MSCs increased the repair effect of MSCs on chemotherapy-induced POF [169]. Additionally, in rats treated with HS-MSCs, levels of sex hormones tended to stabilize [169]. Additionally, low-intensity pulsed ultrasound (LIPUS) can stimulate the expression of a number of growth factors and anti-inflammatory molecules, both of which are important for maintaining follicle growth and preventing GCs apoptosis in the ovary [170, 171]. In a recent study, LIPUS-pretreated human MSCs were found to have additional benefits over naive MSC therapy in rats with chemotherapy-induced POI, including the ability to reduce inflammation, inhibiting granulosa cell apoptosis, repairing ovarian injury, and promoting ovarian function [172].

## Conclusion

In spite of the encouraging outcomes of MSCs therapy in a diversity of diseases, dysfunction of MSCs in host tissue may help explain how some animal studies and clinical trials yield different results. MSCs are vulnerable to the internal environment after infusion, which reduces their survival and grafting to the target tissues [173]. In light of this problem, scientists are exploring different strategies to improve the therapeutic efficacy of MSCs. Recent reports have clarified that pre-conditioning, as a multi-technique approach, could improve MSCs' survival and migration to the target tissue and also could potentiate their immunoregulatory, differentiation, and pro-angiogenic competencies post-transplantation. Nonetheless, there still exist several difficulties in defining the optimal approaches for pre-conditioning in MSC‐based treatment. The compounds used may have negative effects on the cell. The optimal dose of these substances should be determined. It must be ensured that these cells do not undergo abnormal genetic changes. Further, it is also possible to increase the therapeutic effects of MSCs by using combined treatments. Lastly, detailed mechanisms are required to be studied as no simple regulative route protects MSCs from damage.

## Data Availability

Not applicable.

## Abbreviations

MSCs: Mesenchymal stem/stromal cells
BM: Bone marrow
UC: Umbilical cord
AT: Adipose tissue
IDO: Indoleamine 2,3-dioxygenase
LF: Liver failure
VEGF: Vascular endothelial growth factor
FGF: Fibroblast growth factors
TNF-α: Tumor necrosis factor alpha
IFN-γ: Interferon gamma
HIF-1α: Hypoxia-inducible factor 1α
